# Unilateral Lung Agenesis and Left Isomerism in a Neonate: A Case Report of Multi-system Anomalies

**DOI:** 10.7759/cureus.88241

**Published:** 2025-07-18

**Authors:** Trisha G Mukherjee, Kyle E Thurmann, Randy Richardson

**Affiliations:** 1 School of Medicine, Creighton University School of Medicine, Phoenix, USA; 2 Department of Radiology, Creighton University School of Medicine, Phoenix, USA

**Keywords:** cardiac imaging, congenital heart defect, heterotaxy syndrome, left isomerism, lung agenesis, polysplenia, sonic hedgehog signaling

## Abstract

Unilateral agenesis of the lung (UAL) is a rare congenital anomaly resulting from the failed development of the lung bud between weeks three and seven of gestation. It is frequently associated with anomalies involving the cardiovascular, gastrointestinal, and skeletal systems. We describe a full-term male neonate who presented with severe respiratory distress and was found to have complete agenesis of the left lung, bronchus, and pulmonary artery and vein. Additional findings included polysplenia, a midline liver, leftward gallbladder, bifid right thumb, and congenital heart defects, raising concern for an underlying laterality defect. Although heterotaxy syndrome was not formally diagnosed, the constellation of findings is most consistent with left isomerism. Trio-based exome sequencing was negative for pathogenic or likely pathogenic variants, and variants of uncertain significance were not reported. The presence of a bifid thumb, not typically associated with heterotaxy, suggests a broader developmental disruption. Based on established roles in left-right axis patterning, pulmonary morphogenesis, and limb development, the Sonic hedgehog signaling pathway is proposed as a potential contributor. While speculative, this hypothesis provides a developmental framework for the observed anomalies. Early identification of such patterns may aid in diagnosis, prognostication, and coordination of multidisciplinary care. Further research is needed to clarify the embryologic and genetic underpinnings of these rare and complex presentations.

## Introduction

Unilateral agenesis of the lung (UAL) is a rare congenital anomaly resulting from the failed development of the lung bud between weeks three and seven of gestation. The estimated incidence is one per 100,000 live births, with the left lung affected more frequently than the right [1]. While the exact etiology remains unclear, its frequent association with cardiovascular, gastrointestinal, and skeletal anomalies suggests a shared developmental origin involving early embryologic signaling pathways [2]. Despite its rarity, UAL carries significant morbidity and mortality, with respiratory insufficiency and associated anomalies influencing clinical outcomes [3].

Laterality defects are congenital anomalies resulting from disruptions in left-right axis patterning during embryogenesis, leading to abnormal symmetry or positioning of thoracoabdominal organs [4]. These defects can affect the heart, lungs, liver, spleen, and other midline structures [5]. Left isomerism, a subtype of heterotaxy syndrome, is characterized by bilateral left-sidedness and commonly presents with polysplenia, a midline liver, and congenital heart defects [6]. When UAL occurs alongside such features, it may reflect a coordinated embryologic disruption rather than isolated defects affecting individual organs.

In this report, we describe a neonate with left lung agenesis and polysplenia, as well as a midline liver, leftward gallbladder, bifid thumb, and congenital heart defects. To our knowledge, the co-occurrence of these anomalies, particularly UAL, features of left isomerism, and a skeletal malformation, has been rarely reported and may suggest a broader developmental pattern. We hypothesize that these anomalies may result from disruption of the Sonic hedgehog (Shh) signaling pathway, which has been shown in animal models to regulate left-right axis formation, lung morphogenesis, and limb development [7-9]. This case highlights the need for further investigation into the genetic and embryologic mechanisms underlying UAL and whether its co-occurring anomalies represent an isolated occurrence or a manifestation of a broader laterality disorder.

## Case presentation

A male neonate, weighing 2.92 kg, was delivered at a tertiary medical center via elective repeat cesarean section at 39 weeks and one day of gestation to a 36-year-old, gravida three para two, mother with a history of prior uterine surgery. The pregnancy was complicated by advanced maternal age and group B *Streptococcus* (GBS) positivity, for which the mother received cefazolin prophylaxis. A prenatal ultrasound failed to visualize the fetal heart, prompting a referral for fetal echocardiography, but the follow-up was not completed for unknown reasons.

At birth, the infant exhibited severe respiratory distress, bradycardia (heart rate <100 beats per minute), and cyanosis, with Apgar scores of one at one minute, one at five minutes, and six at 10 minutes. Positive pressure ventilation via bag-mask was initiated; however, due to persistent hypoxia and cyanosis, the infant required endotracheal intubation and mechanical ventilation, which resulted in only mild improvement in oxygenation. A chest X-ray performed in the delivery room demonstrated complete opacity of the left lung field with an elevated gastric bubble, initially raising concern for congenital diaphragmatic hernia (CDH) or atelectasis.

Upon neonatal intensive care unit admission, the infant was transitioned to high-frequency oscillatory ventilation due to persistent hypoxia but did not tolerate it well. Curosurf was administered via endotracheal tube; however, given the underlying structural anomaly, the response was minimal. Empiric antibiotics with ampicillin and gentamicin were initiated due to maternal GBS positivity, but blood cultures were negative, which led to the discontinuation of antibiotics. A chest X-ray revealed a pneumothorax, prompting chest tube placement. A follow-up X-ray confirmed resolution of pneumothorax, but the patient remained critically dependent on ventilatory support.

**Figure 1:**
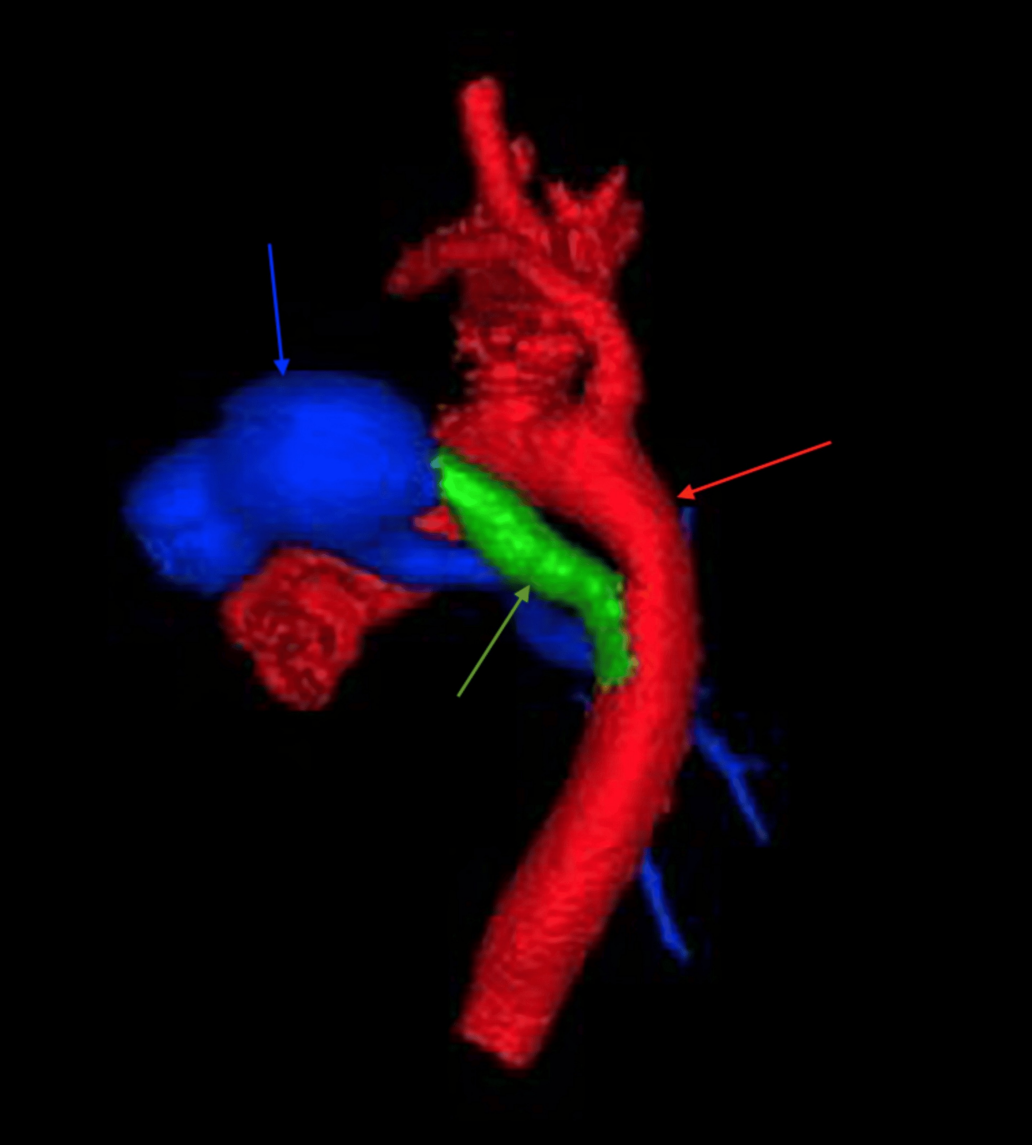
Three-dimensional computed tomography angiography reconstruction demonstrating patent ductus arteriosus (green arrow) connecting pulmonary artery (blue arrow) and aorta (red arrow)

**Figure 2:**
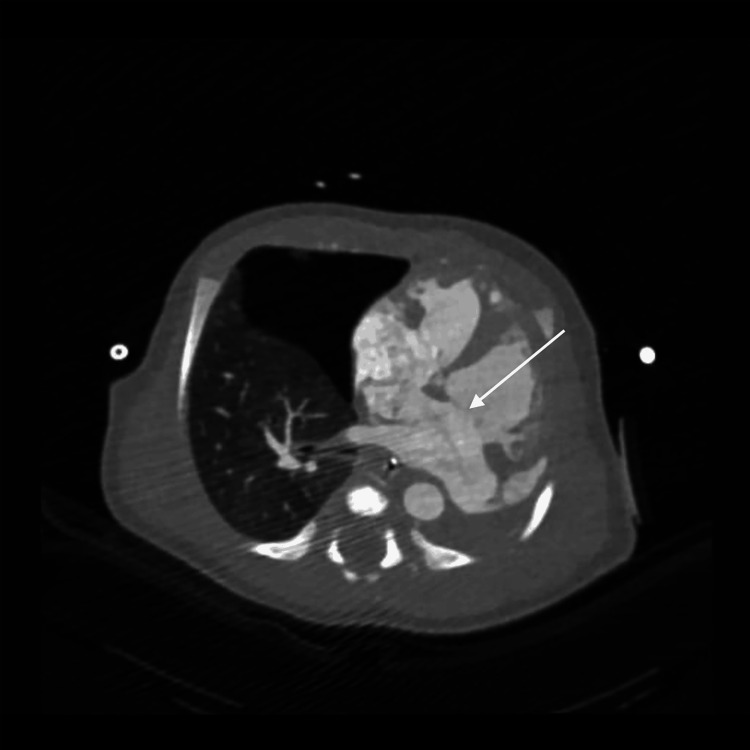
Axial computed tomography scan demonstrating atrial septal defect (white arrow) between right and left atria

**Figure 3:**
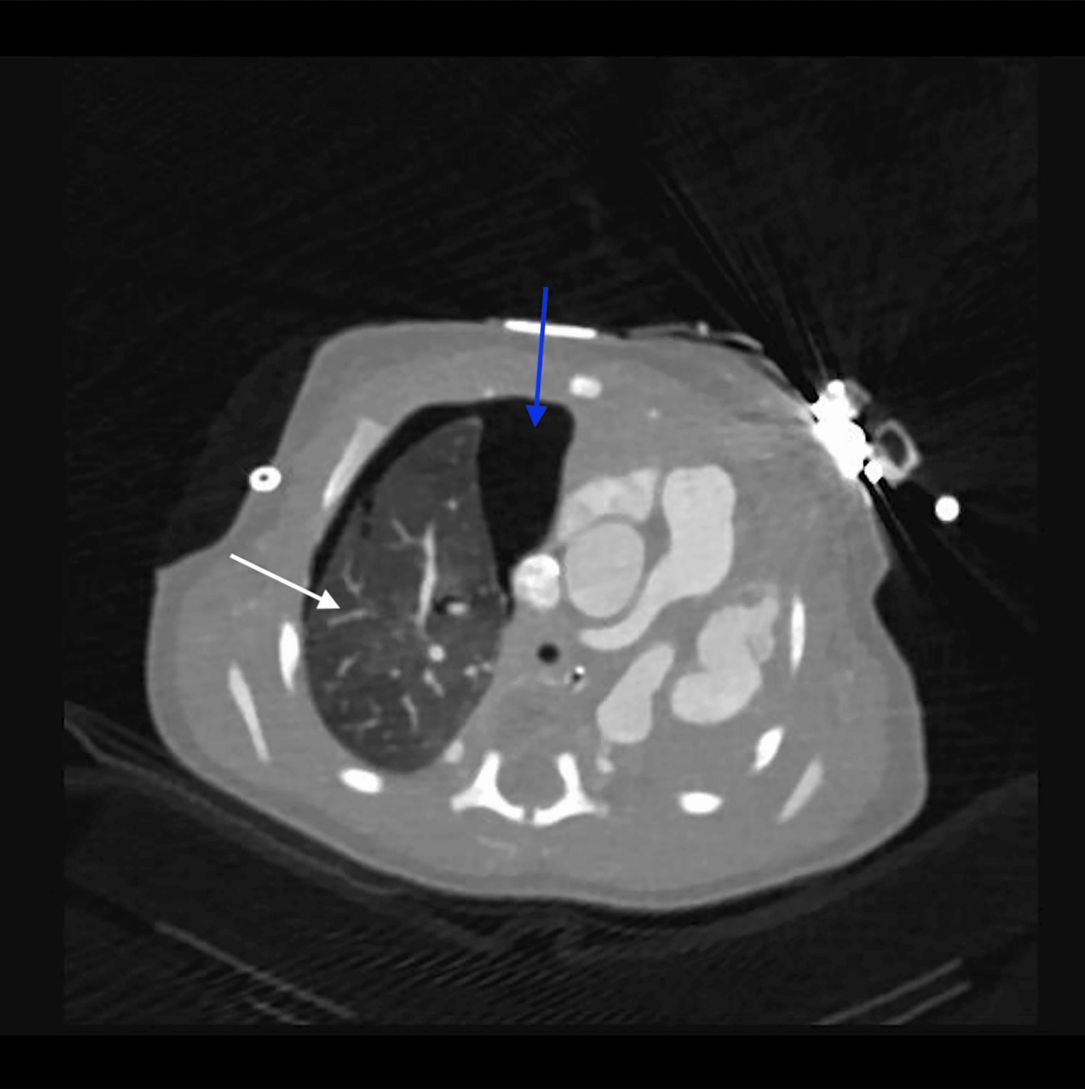
Axial computed tomography scan demonstrating complete absence of the left lung with a shift of the mediastinum to the left The right lung is indicated by the white arrow a right-sided pneumothorax is present (blue arrow).

**Figure 4:**
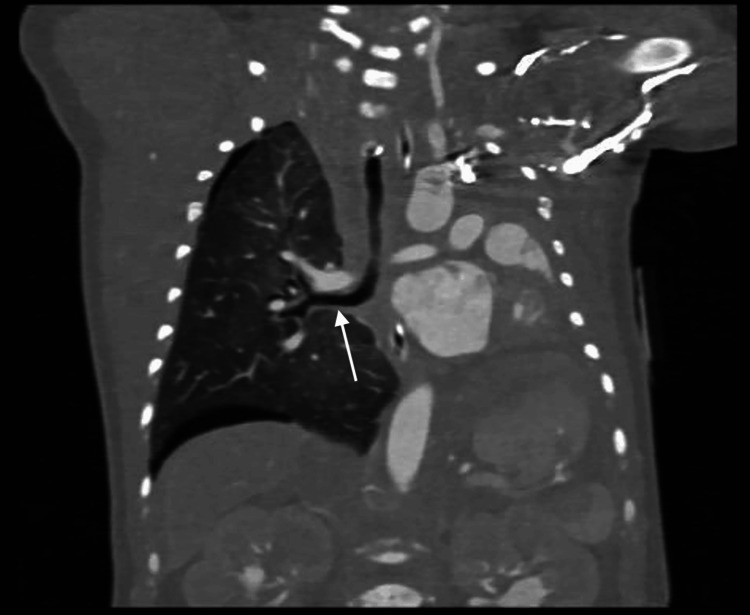
Coronal computed tomography scan demonstrating left lung agenesis and leftward mediastinal shift The left main bronchus is absent, and only the right main bronchus is visualized (white arrow).

**Figure 5:**
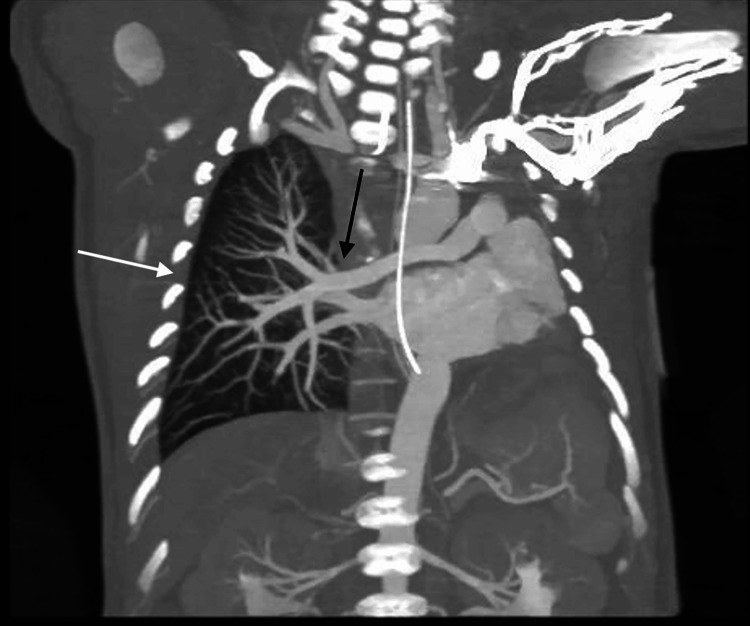
Coronal computed tomography angiography demonstrating left lung agenesis with absent pulmonary arteries and veins The right lung (white arrow) is expanded and receives the entire pulmonary circulation (black arrow).

**Figure 6:**
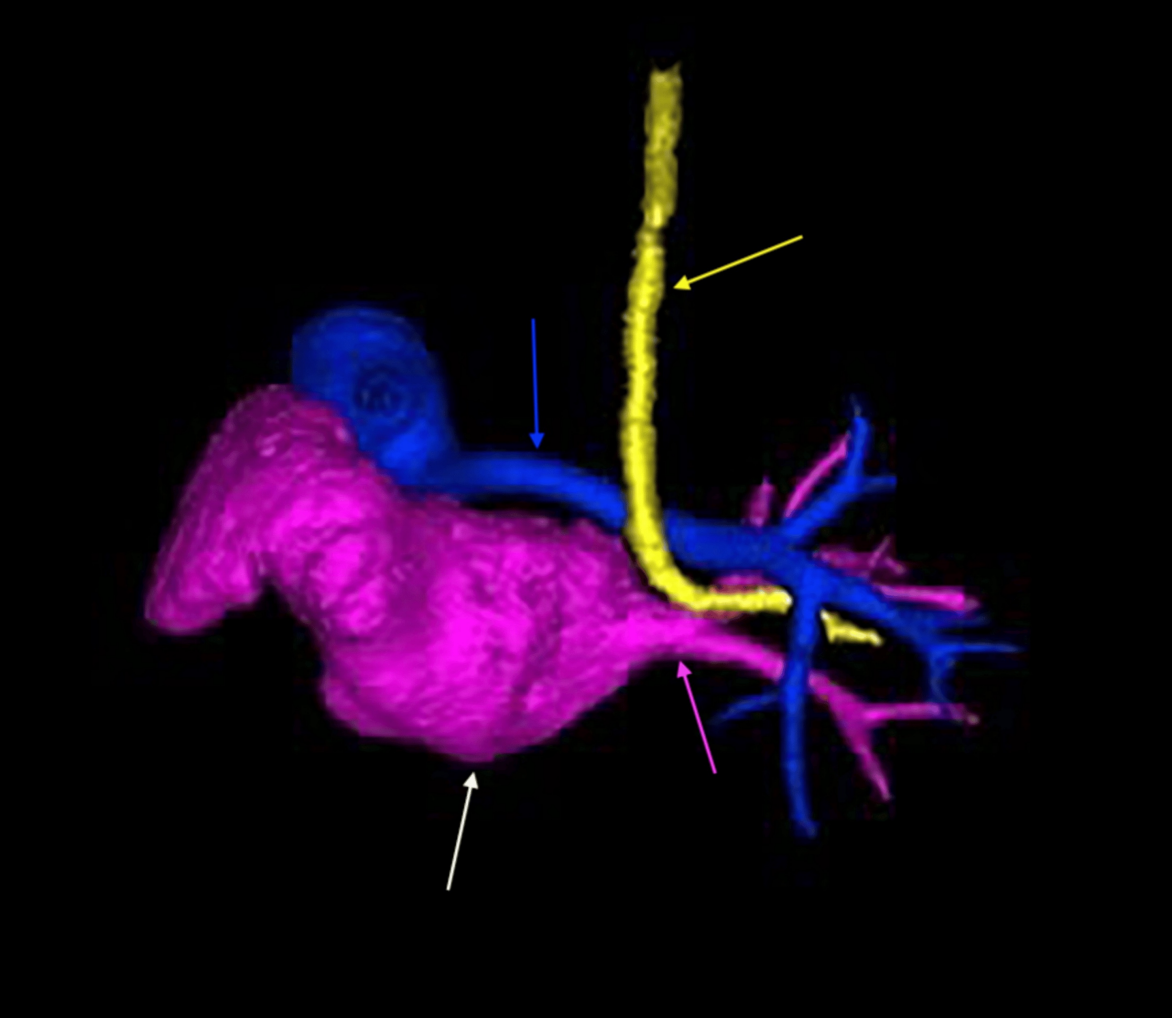
Three-dimensional computed tomography reconstruction demonstrating complete agenesis of the left lung with absence of the left pulmonary vasculature Only the right pulmonary vasculature is visualized: the right pulmonary vein (pink arrow) and right pulmonary artery (blue arrow). The heart is indicated by the white arrow, and the trachea is deviated (yellow arrow).

Cardiac imaging revealed several congenital abnormalities. An echocardiogram identified a large patent ductus arteriosus (PDA), small atrial septal defect (ASD), and right ventricular pressure at systemic levels, with no visualization of the left pulmonary artery. These findings, along with further anatomic detail, were visualized on computed tomography (CT) (Figures 1, 2). CT also confirmed left lung agenesis, absence of the left pulmonary artery and bronchus, and no visualized left pulmonary veins. The right pulmonary artery and lung appeared hypoplastic, and no evidence of CDH was seen (Figures 3-6).

**Figure 7:**
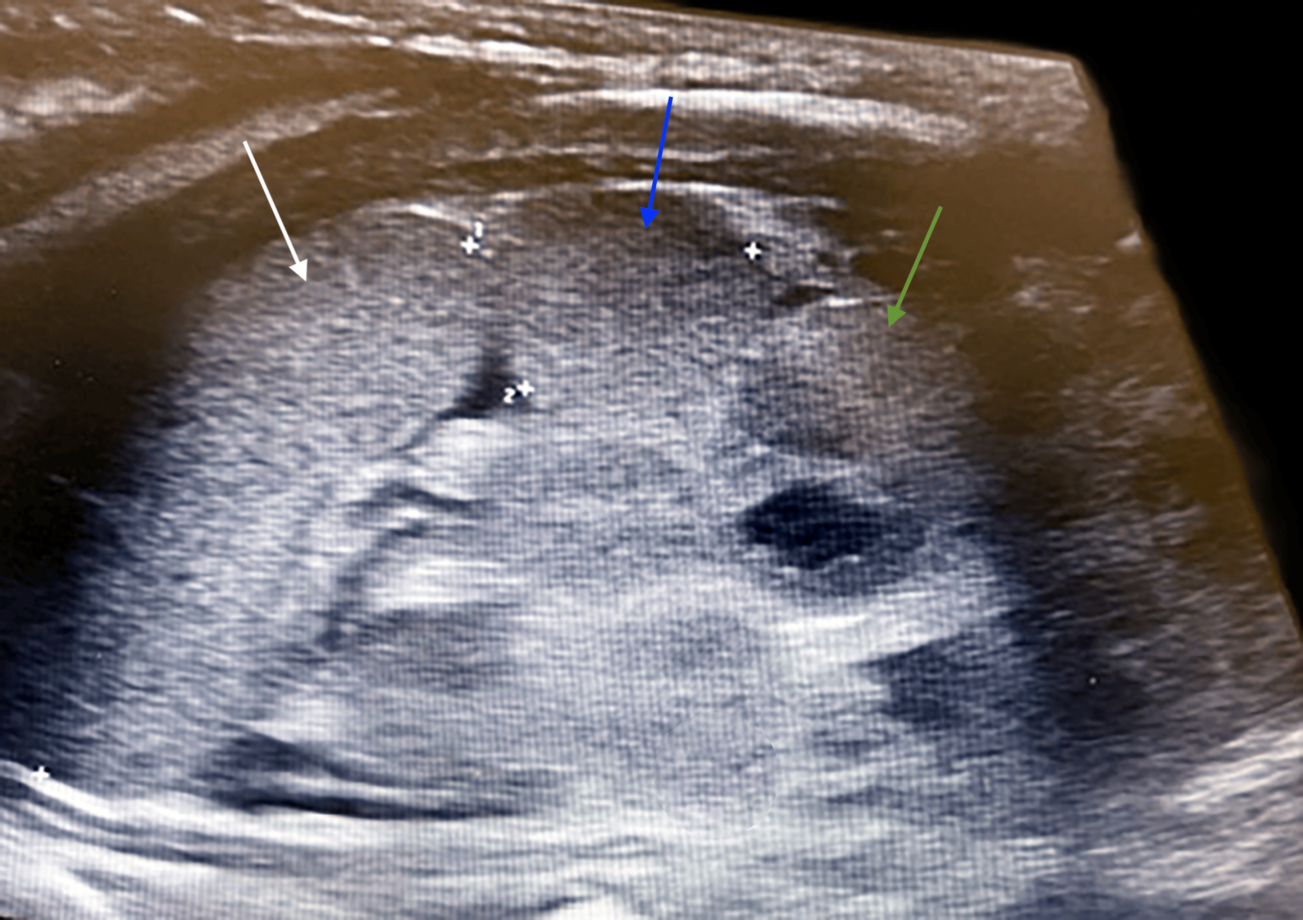
Longitudinal ultrasound view demonstrating polysplenia (white, blue, and green arrows)

**Figure 8:**
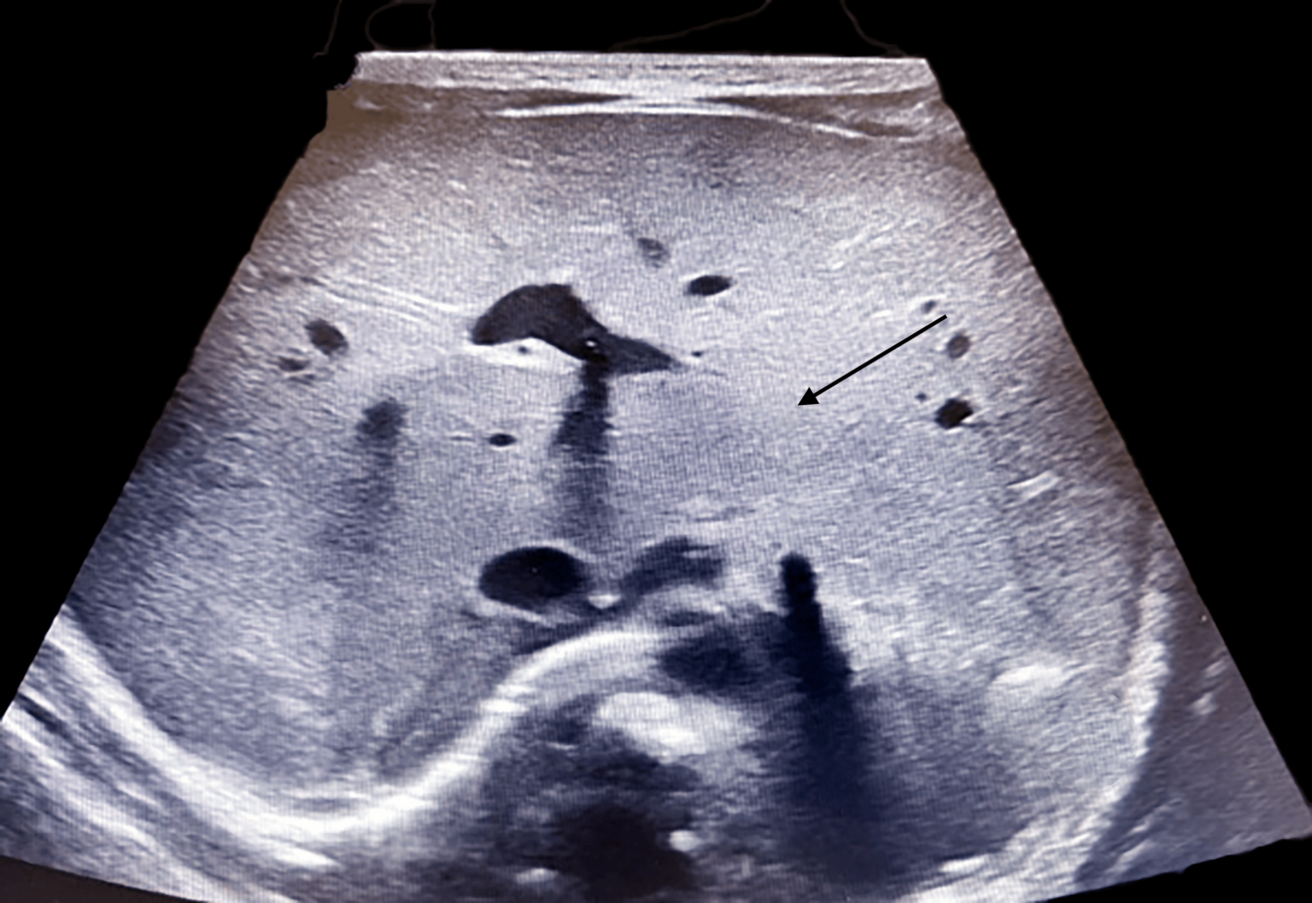
Transverse ultrasound view of the upper abdomen showing a liver extending across the midline (black arrow)

**Figure 9:**
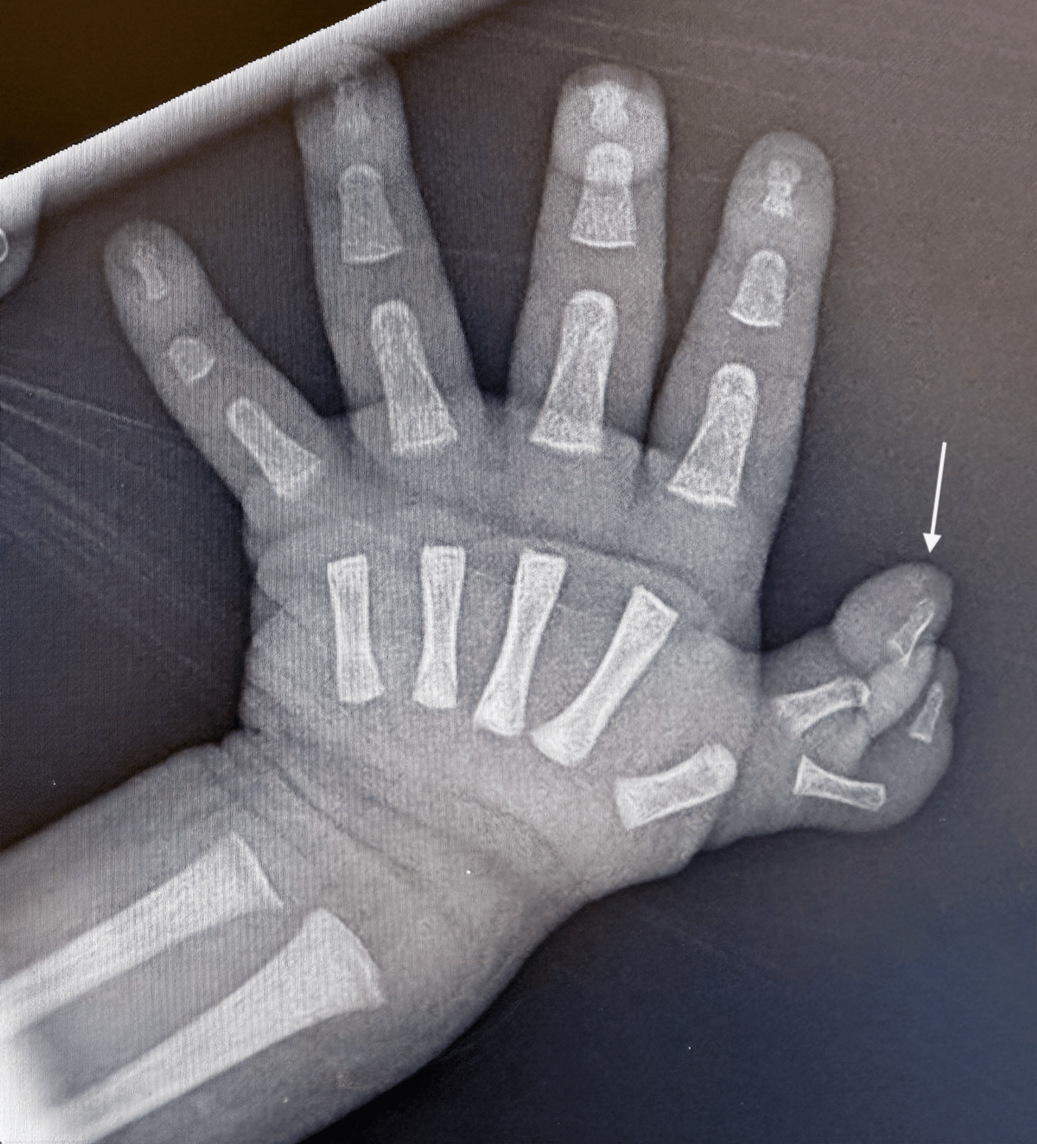
Anterior-posterior radiograph of the right hand demonstrating a bifid thumb (white arrow)

Further imaging identified additional congenital anomalies. An abdominal ultrasound revealed polysplenia (Figure 7), a midline liver (Figure 8), and a leftward gallbladder. A renal ultrasound demonstrated mild left-sided central calyceal and pelvic fullness. A hand X-ray confirmed a bifid right thumb (Figure 9). Genetic testing included trio-based exome sequencing, which was negative for pathogenic or likely pathogenic variants. Variants of uncertain significance were not reported.

The infant required continued ventilatory support due to persistent respiratory insufficiency. By day nine of life, he was extubated and transitioned to nasal intermittent positive pressure ventilation. On day 21, he was transitioned to a low-flow nasal cannula. At discharge (day 29 of life), the infant remained on home oxygen therapy with pulse oximetry monitoring, as prior attempts to wean were unsuccessful due to desaturation. Follow-up with pediatric cardiology was scheduled to monitor the ASD and right pulmonary artery hypoplasia. A pulmonary follow-up was scheduled for two months to reassess oxygen dependency and pulmonary function. Additional follow-ups included genetics for further evaluation of congenital anomalies and immunology to assess potential polysplenia-related immune dysfunction. A primary care follow-up was scheduled shortly after discharge to monitor growth, development, and feeding progress.

## Discussion

UAL is a rare congenital anomaly caused by the failed development of the primitive foregut lung buds [1]. When this developmental process is completely disrupted, no lung tissue, bronchial structures, or pulmonary vasculature form [1]. In this case, the patient was diagnosed with type 1 UAL, characterized by complete agenesis of the left lung, bronchus, and pulmonary vasculature, as confirmed by CT imaging [1,2].

The patient also presented with polysplenia, a midline liver, a leftward gallbladder, a bifid thumb, and congenital heart defects, which raises concern for an underlying laterality defect involving left-right axis development. These findings share significant overlap with the clinical features of heterotaxy syndrome, particularly left isomerism (polysplenia syndrome), which is typically characterized by multiple spleens, a midline liver, and structural cardiac anomalies [6]. The addition of UAL, a rare extracardiac manifestation of heterotaxy, contributes to the diagnostic complexity of this presentation [6].

While the visceral anatomy is most indicative of left isomerism, the cardiac findings are also supportive. This patient was found to have an ASD and a PDA, both commonly reported in heterotaxy syndrome [6]. In addition, imaging revealed the complete absence of the left pulmonary vein. While left isomerism is often associated with total or partial anomalous pulmonary venous return, the absence of a pulmonary vein altogether appears to be an unusual or potentially unreported variant [4,6]. 

Although a formal diagnosis of heterotaxy syndrome was not made, this patient’s constellation of findings meets clinical criteria for left isomerism. Polysplenia, a midline liver, leftward gallbladder, and structural heart defects, including ASD, PDA, and abnormal pulmonary venous anatomy, are hallmark features of this subtype [4,6]. Left isomerism is clinically diagnosed when thoracoabdominal organ arrangement deviates from situs solitus or situs inversus and exhibits symmetry or left-sided duplication [4,6]. The presence of multiple left-sided features in this case supports the diagnosis of left isomerism; however, the patient also exhibited a bifid thumb, a skeletal defect not commonly associated with heterotaxy [10]. While limb abnormalities are not typically part of the heterotaxy spectrum, their presence in this case suggests a broader developmental disruption beyond thoracoabdominal organ patterning, potentially involving early signaling pathways such as Shh. 

The Shh signaling pathway plays a central role in embryologic processes essential to left-right axis formation, pulmonary morphogenesis, and limb development [7]. Other genetic mechanisms have also been implicated in heterotaxy syndrome, including  *ZIC3*, *NODAL*, *LEFTY2*, *ACVR2B*, *CFC1*, *CITED2*, and *GDF1*, which primarily disrupt nodal flow and downstream transcriptional regulation, resulting in laterality defects and cardiac anomalies; however, their involvement in skeletal development is less established [6]. 

Notably, experimental studies of Shh null mice demonstrate phenotypes strikingly similar to those observed in our patient, including left atrial isomerism, atrioventricular septal defect, and absent pulmonary veins [11]. Shh influences both left-right patterning and second heart field contributions, particularly at the venous pole, which is essential for proper atrioventricular septation [9,11]. In addition, Shh is a critical morphogen in embryonic lung development, where it regulates epithelial-mesenchymal interactions that guide airway branching and alveolar formation [7]. In limb development, Shh acts as a morphogen that specifies anterior-posterior axis identity; disruptions in its concentration or timing can result in bifid or duplicated digits [12]. The presence of a bifid thumb in this case, therefore, extends the phenotypic spectrum beyond classic heterotaxy and suggests a broader disturbance in mesodermal patterning [6,12]. Taken together, these findings support the hypothesis that the anomalies in this case may result from upstream disruption of Shh signaling or dysregulation of related developmental fields governing cardiac, pulmonary, and limb morphogenesis [7,9,11,12].

This case presents several diagnostic limitations. Although trio exome sequencing was performed and returned negative for pathogenic or likely pathogenic variants, no information was available regarding variants of uncertain significance, limiting interpretation. Such variants may have involved pathways like Shh that remain unclassified and could explain the constellation of anomalies observed. No defined monogenic syndrome was diagnosed, and heterotaxy syndrome was not formally recognized in the patient’s clinical documentation. The diagnosis of left isomerism was made retrospectively based on postnatal imaging findings, including polysplenia, midline liver, leftward gallbladder, and cardiac anomalies. In addition, fetal echocardiography was recommended during prenatal care due to poor visualization of the heart, but the patient’s mother did not follow up, limiting early detection of cardiac or laterality anomalies. While no teratogenic exposures were documented during pregnancy, potential environmental disruptions during early embryogenesis cannot be fully excluded and may warrant further investigation in similar cases.

Following birth, the patient’s clinical course was dominated by severe respiratory distress due to left lung agenesis, which appropriately prioritized pulmonary stabilization and may have delayed a more comprehensive evaluation of systemic anatomy. Management was primarily supportive and aimed at optimizing respiratory function. The patient required escalating ventilatory support, including high-frequency oscillatory ventilation and assisted control ventilation, before transitioning to non-invasive positive pressure ventilation and nasal cannula oxygen therapy. No surgical interventions were pursued during the neonatal period. The patient was discharged on home oxygen, with outpatient follow-up arranged with pulmonology, cardiology, genetics, and immunology. These factors collectively limited timely recognition of the underlying pattern of anomalies and constrained definitive genetic or syndromic classification. Long-term prognosis remains uncertain and will depend on pulmonary function, infection risk, and the evolution of associated cardiac anomalies [6]. Continued multidisciplinary monitoring will be essential to guide future care and assess developmental outcomes [6,13].

## Conclusions

This case highlights a rare and complex presentation of left UAL with multiple co-occurring anomalies, including polysplenia, midline liver, congenital heart defects, and a bifid thumb. While a definitive diagnosis of heterotaxy syndrome was not established clinically, the constellation of findings is most consistent with left isomerism. The absence of identified pathogenic genetic variants, along with the breadth of anomalies across multiple organ systems, suggests a broader disruption of early embryologic signaling pathways, with Shh signaling as a plausible contributor. Although this connection remains hypothetical, it provides a useful framework for understanding the co-occurrence of cardiac, pulmonary, and skeletal abnormalities. This case underscores the diagnostic and management challenges posed by rare congenital anomalies and highlights the importance of multidisciplinary follow-up. Further investigation into the developmental pathways underlying such presentations is needed to improve diagnosis, prognostication, and counseling in similar cases.
